# Genome-wide insights into population structure and genetic history of tunisian local cattle using the illumina bovinesnp50 beadchip

**DOI:** 10.1186/s12864-015-1638-6

**Published:** 2015-09-04

**Authors:** Slim Ben Jemaa, Mekki Boussaha, Mondher Ben Mehdi, Jun Heon Lee, Seung-Hwan Lee

**Affiliations:** National Institute of Agronomic Research of Tunisia, Laboratoire des Productions Animales et Fourragères, Rue Hédi Karray, 2049 Ariana, Tunisia; INRA, UMR1313, Unité Génétique Animale et Biologie Intégrative, Domaine de Vilvert, F-78352 Jouy-en-Josas, France; AgroParisTech, UMR1313, Unité Génétique Animale et Biologie Intégrative, Domaine de Vilvert, F-78352 Jouy-en-Josas, France; Livestock and Pasture Office, 1002 Tunis Belvedere, Tunisia; Department of Animal Science and Biotechnology, Chungnam National University, Daejeon, 305-764 South Korea; Hanwoo Experiment Station, National Institute of Animal Science, RDA, Pyeongchang, 232-952 South Korea

**Keywords:** Cattle, SNP, Genetic structure, Admixture

## Abstract

**Background:**

Tunisian local cattle populations are at risk of extinction as they were massively crossed with imported breeds. Preservation of indigenous livestock populations is important because each of them comprises a unique set of genes resulting from a local environment-driven selection that occurred over hundreds of years. The diversity and genetic structure of Tunisian local cattle populations are poorly understood. However, such information is crucial to the conservation and sustainable use of genetic resources.

In addition, comparing the genomic structure of population sets from different parts of the world could help yield insight into their origin and history.

In the present study, we provide a detailed assessment of the population structure of the three Tunisian local cattle populations using various methods, and we highlight their origin and history by investigating approximately ~38,000 SNPs in a broad panel of 878 individuals from 37 worldwide cattle breeds representative of African, European and indicine populations.

**Results:**

Our study revealed a low level of divergence and high genetic diversity in Tunisian local cattle reflecting low levels of genetic drift. A Comparison with the worldwide cattle panel pinpointed the admixed origin of the genome of the three Tunisian populations with the two main European and African ancestries. Our results were in agreement with previous historical and archaeological reports about the past gene flow that existed between North African and South European breeds, in particular with Iberian cattle. We also detected a low-level indicine introgression in the three Tunisian populations and we inferred that indicine ancestry was inherited via African ancestors.

**Conclusions:**

Our results represent the first study providing genetic evidence about the origin and history of Tunisian local cattle. The information provided by the fine-scale genetic characterization of our study will enhance the establishment of a national conservation strategy for these populations. These results may enable the identification of genetic variants involved in adaptation to harsh environmental conditions.

**Electronic supplementary material:**

The online version of this article (doi:10.1186/s12864-015-1638-6) contains supplementary material, which is available to authorized users.

## Background

During the last century, an erosion of cattle genetic resources was observed in many countries around the world as a result of a massive replacement of low-productive local breeds with highly productive ones. According to the FAO (Food and Agriculture Organisation), there are currently more than 1300 taurine and indicine breeds in the world. Among these, many local cattle breeds are already extinct (16 %), endangered (16 %) or have an as-yet unknown risk status (30 %), and they may disappear before they are fully studied or their characteristics are recorded [1]. Many of these breeds have been selected for their adaptation to local environments, as well as from recent artificial selection programs, thus leading to a wide range of inter-population genetic diversities. The conservation of local breeds and the monitoring of their genetic diversity are fundamental to meet future breeding needs, especially in the context of global climate change [2, 3].

The emergence of high-throughput SNP genotyping facilities coupled with the gradual reduction of genotyping costs may help elucidate the genetic diversity and structure of endangered populations. Such information is crucial for the conservation and sustainable use of genetic resources. Moreover, comparing the genomic structure of population sets from different parts of the world provides a new glimpse into their origin and history.

Local cattle in Tunisia belong to three main populations according to morphological criteria: the Blonde du Cap Bon (BLCAP), the Brune de l’Atlas Grise (BRATG), and the Brune de l’Atlas Fauve (BRATF). Today, these three populations are endangered by massive crossing with imported breeds to improve their production performance [4, 5]. Indigenous cattle in Tunisia play an important role as cash reserves in low-income extensive farming systems in the arid regions of the center as well as in the northern mountainous regions of the country. These animals have low maintenance costs and are well adapted to the harsh conditions of these regions (reduced food resources, elevated temperatures during the hot season, abundance of parasites and pathogens). Additionally, local cattle have a strong cultural significance as they reflect a long history of symbiosis with human populations.

The aim of the present study was to provide a detailed assessment of the genetic structure of Tunisian local cattle populations and to trace back their origin and history using medium density SNP chips.

For this purpose, we first genotyped 50 Tunisian individuals from the three populations using the Illumina BovineSNP50 BeadChip, and we subsequently combined our genotyping data with publicly available data from 34 other populations (bovine Hapmap dataset) representative of African (AFT), European (EUT) and zebu (ZEB) cattle. The results of our study provide a detailed assessment of the genetic diversity and structure of Tunisian local cattle populations, shedding light on the origin and history of Tunisian local populations through their genetic relationship with the bovine Hapmap dataset populations; these insights into population structure and genetic diversity in Tunisian local populations are further elucidated by relating our results to previous reports about the domestication and dispersal of cattle in Africa and Europe.

## Results

### Genetic diversity

The genetic diversity of Tunisian breeds measured by expected heterozygosity (Hs) using all available BovineSNP50 BeadChip SNPs was similar to that observed for European breeds and broadly equivalent to 0.32. Indicine populations of Indian origin (GIR, BRA, BRM and NEL) had the lowest Hs rates, ranging from 0.16 to 0.2, while African populations had slightly higher Hs values ranging from 0.19 to 0.24 (Additional file 1). The large difference in Hs levels between European breeds the one hand and African and indicine populations on the other is most likely due to the fact that SNPs on the BovineSNP50 BeadChip were primarily identified from European breeds. Bearing this in mind, an excess of low MAF SNPs (<0.1) was observed within African and indicine breeds (Additional file 2). The exclusion of SNPs with MAF lower than 1 % observed in AFT and ZEB breeds resulted in an increase of the expected Hs levels for all breeds (Additional file 1). This increase was most pronounced within hybrid (AFTXZEB), AFT, and ZEB populations with African origins (ZBO and ZFU). For Tunisian populations, the increase in expected Hs was smaller than that of the hybrid (AFTXZEB), and more pronounced than that observed for European breeds. Therefore, the inference of population structure from the BovineSNP50 BeadChip should be done with caution due to this ascertainment bias.

Average inbreeding for all populations, measured by Fis, was almost null (0.0024) but slightly positive for BRATG and BRATF (0.0149 and 0.0302, respectively) indicating the absence of any cryptic population structure in our Brune de l’Atlas samples (Additional file 1).

Fis was slightly negative for BLCAP (−0.0121) but Fis 95 % CI computed with 5000 randomly chosen SNPs and 100,000 bootstraps was slightly positive (Table S2 in Additional file 3). This could indicate the absence of any population structure for BLCAP, as seen with the Brune de l’Atlas populations. On the other hand, the OUL, BSW and SGT breeds had negative values for both the Fis values and Fis 95 % CI, indicating an excess of Heterozygosity beyond that expected under Hardy-Weinberg equilibrium within these populations.

### Assessment of population structure using tree-based methods

**Fig 1:**
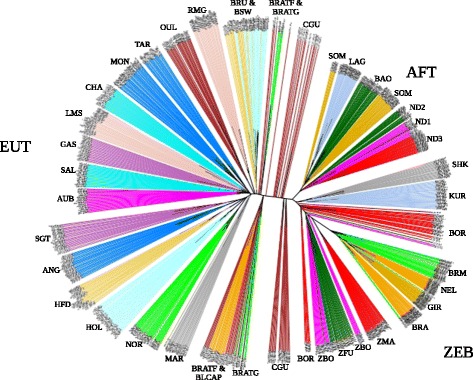
Unrooted neighbor-joining tree of 878 individuals using 38,597 SNPs. The distance between each pair of individuals was defined as the number of loci for which they differ. Edges are colored according to the individual breed of origin

In order to have a genetic characterization of Tunisian local breeds, we first computed the genetic distance between each pair of individuals from the number of loci for which they differ. A neighbor-joining tree was computed based on the estimated genetic distances (Fig. 1). The topology of the tree showed a clear separation between AFT, ZEB and EUT populations (respectively in the upper right, lower right, and the half left circle bounded by Brown Swiss (BRU and BSW) and Maraichine (MAR)). Individuals belonging to the same breed were grouped together leading to a clear separation between most breeds. Most of the Tunisian animals from the three populations were grouped into a single cluster that branched close to the European breeds. Some animals belonging to BRATF and BRATG branched close to BRU and BSW, suggesting a Brown Swiss influence on these animals. The admixed breed OUL (EUTXAFT) from Morocco was placed on the side of European breeds (root near RMG) whereas individuals belonging to the SGT breed (EUTXZEB) branched near ANG. West African hybrid populations (SHK, KUR and most of the BOR animals) were placed between African Longhorn N’Dama (ND1, ND2 and ND3) and indicine populations from Indian origin. Similarly, hybrid CGU individuals (AFTXEUTXZEB) were grouped into two distinct clusters: the first was located in an intermediary position between AFT and EUT populations, while the second branched close to the African Zebu, suggesting an indicine influence (Fig. 1).

### Assessment of population structure using multivariate statistics

**Fig 2:**
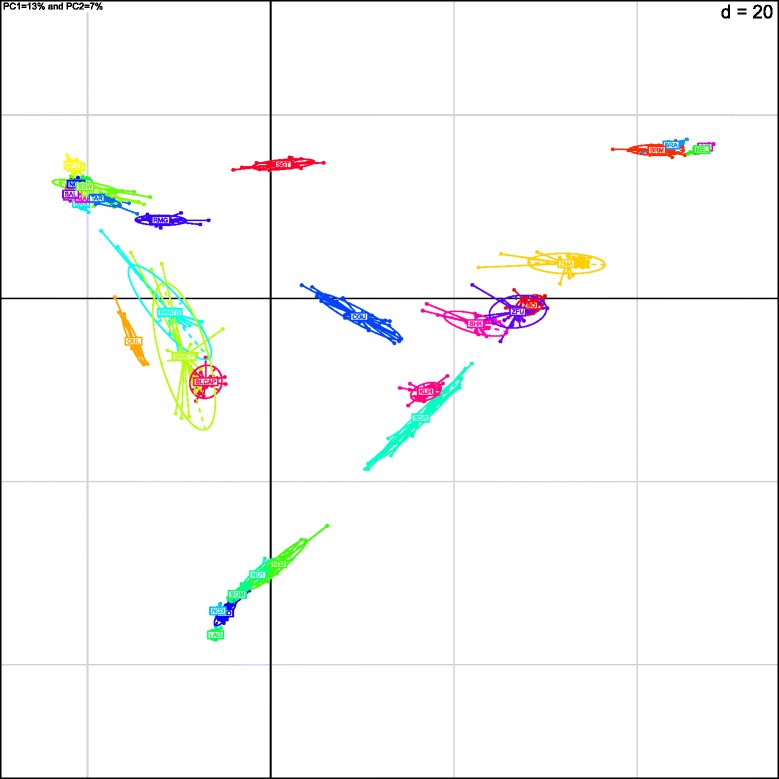
PCA results of allele frequencies obtained from 38,597 SNPs genotyped in 878 cattle individuals from 37 populations. The first two principal components, PC1 and PC2, accounted for 13 % and 7 %, respectively of the total variation. The first PC axis (x-axis) aligned populations according to an indicine/taurine gradient while the second (y-axis) aligned them according to an AFT/EUT gradient

We used Principal Component Analysis (PCA) to locate the Tunisian individuals compared to the other populations of the study. PCA grouped individuals in clusters according to their populations of origin (Fig. 2). The first two principal components (PCs) explained approximately 13 % (PC1) and 7 % (PC2) of the global variation. The first PC axis (x-axis) aligned populations according to an indicine/taurine gradient while the second one (y-axis) aligned them according to an AFT/EUT gradient. Tunisian populations were positioned close to European breeds along the second PC2 axis but slightly tilting toward zebu on PC1, especially for BLCAP. For the other hybrid populations, SGT animals were positioned close to taurines on the PC1 axis and close to EUT breeds on the PC2 axis. Individuals belonging to the CGU breed fell at an intermediate position between indicine and taurine breeds on PC1, but were closer to EUT than AFT populations on PC2. As expected, BOR and KUR animals were placed on the ZEB/AFT segment, closer to indicine breeds on PC1 and to AFT on PC2. Finally, SHK, which was originally ranked as an AFT breed, was placed on the ZEB/AFT segment but closer to indicine than AFT.

The predefined group DAPC (see methods) gave similar results to those obtained with PCA, i.e., a first indicine/taurine axis and a second AFT/EUT axis (Additional file 4). However, DAPC enabled better discrimination between breeds from the same origin. In this regard, Northern European breeds (NOR, HOL, HFD and ANG) were clearly separated from the other EUT breeds. African Shorthorn (LAG) was also clearly separated from the other AFT populations.

DAPC provides membership probabilities of each individual for the different populations of the study based on the retained discriminant functions. Plotting these probabilities for Tunisian animals revealed that one individual originally identified as BRATF was instead assigned to BRU (Additional file 5). This individual was therefore discarded from our data analysis. In the same manner, four individuals initially identified as BLCAP and five individuals initially identified as BRATG were assigned to the BRATF population with membership probability close to one (Additional file 5). We thus corrected for the population origin of these animals in our data analysis.

In the a posteriori DAPC (see methods), the grouping obtained for k = 15 gave an accurate description of the data (Additional file 6). Most of the Tunisian individuals were assigned to cluster eight and three BRATG individuals to cluster 10. Both groups have their center of gravity tightly linked. Genetic clusters 12 (South European breeds), nine (Brown Swiss) and four (OUL and RMG breeds) were the closest to Tunisian individuals.

### Assessment of population structure using model-based methods

**Fig 3:**
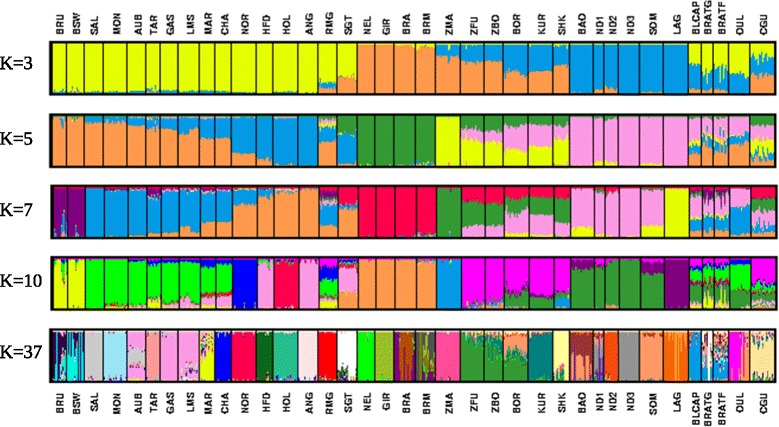
Unsupervised hierarchical clustering of the 877 individuals from the 37 populations. Results for k (number of clusters) = 3, k = 5, k = 7, k = 10 and k = 37 are shown. Individuals are grouped by population. Each individual is represented by a vertical bar. The proportion of the bar in each of k colors corresponds to the average posterior likelihood that the individual is assigned to the cluster indicated by that color. Populations are separated by black lines

We used the unsupervised hierarchical clustering implemented in the Admixture software [6] to estimate proportions of ancestry from each contributing genetic cluster in the populations of the study (Fig. 3). In this analysis, we used several values of k corresponding to ancestral populations.

For k equal to 3, the AFT, EUT and ZEB populations were clearly separated, whereas hybrid populations showed varying proportions of African, European and indicine ancestries. The Tunisian samples were made up of the three ancestries in different proportions. In particular, BLCAP individuals showed the greatest proportion of AFT (49 %) ancestry (41 % EUT and 10 % ZEB) followed by BRATF with 43 % of AFT (49 % of EUT and 8 % of ZEB ancestries) and by BRATG with 32 % of AFT (60 % of EUT and 8 % of ZEB ancestries). Among all African taurines only SHK showed more indicine (57 %) than African (39 %) ancestry.

Increasing the number of clusters to k = 5 resulted in the main subgroups of European breeds. The first group included Brown Swiss (BRU and BSW) and South European breeds (SAL, MON, AUB, TAR, GAS, LMS) while the second one can be viewed as North European breeds (ANG, HOL, HFD). On the other hand, Tunisian populations showed more Southern than Northern European ancestry. Of the three populations, BRATG showed the highest Southern European admixture level (~45 %) while BLCAP showed the lowest (~26 %).

When k was set to 7, the two Brown Swiss populations, BRU and BSW, formed a distinct cluster while two subdivisions appeared among African taurines that might be interpreted as representative of Longhorn (ND1, ND2, ND3) and shorthorn (LAG). Interestingly, both BRATF and BRATG populations showed a substantial level of admixture with Brown Swiss (~11 % for BRATF and ~17 % for BRATG) and up to 25 % of admixture has been observed with some animals. African ancestry within Tunisian populations and OUL (a North African breed), is mainly from Longhorn (on average, 42 %, 36 %, 26 % and 36 % for BLCAP, BRATF, BRATG and OUL, respectively).

At k = 10, ZBO and ZFU were placed together in a separate cluster. Tunisian populations appear to have inherited their indicine ancestry via African ancestors since they shared almost all of their indicine ancestry with the ZBO/ZFU cluster.

Finally, fixing k to 37 (the number of populations used in this study) revealed that almost 85 % of the BLCAP genome was shared with 35 % and 26 % of the BRATF and BRATG genomes, respectively.

### Genetic differentiation

To get more insight into the genetic relationship between the different populations of the study, we estimated the Fst for pairwise comparisons of all populations, using both Genepop software (Table S3 in Additional file 3) and the R package StAMPP (Table S2 in Additional file 3). The latter allows for the computation of Fst 95 % CI intervals (5000 bootstraps).

Pairwise Fst values obtained with both software packages were very similar and revealed lower values for BLCAP/BRATF (0.0246) and BRATF/BRATG (0.0375) but higher levels of differentiation between BLCAP and BRATG (0.0638). Tunisian populations had moderate Fst values (i.e., <=0.1) with almost all South European breeds and presented high levels of differentiation (i.e., Fst > 0.1) with North European breeds. We also observed moderate Fst values between Brune de l’Atlas populations and the BRU breed.

Although, high Fst values were observed between Tunisian populations and African cattle, these were clearly lower with African Longhorn than African shorthorn populations (LAG). Among the three Tunisian populations, BRATF had the lowest Fst values with both African and European populations, while BLCAP tends to have lower levels of differentiation with African populations than BRATG. This trend was reversed when comparing pairwise Fst values of these two breeds with South European breeds (Additional file 7).

Interestingly, low to moderate Fst values were observed between Tunisian populations and CGU ranging from 0.0501 for BRATF/CGU to 0.0801 for BRATG/CGU. Surprisingly, these values were higher than those observed between Tunisian populations and OUL (ranging from 0.0687 for BRATF/OUL to 0.0993 for BRATG/OUL).

### Phylogenetic analysis

**Fig 4:**
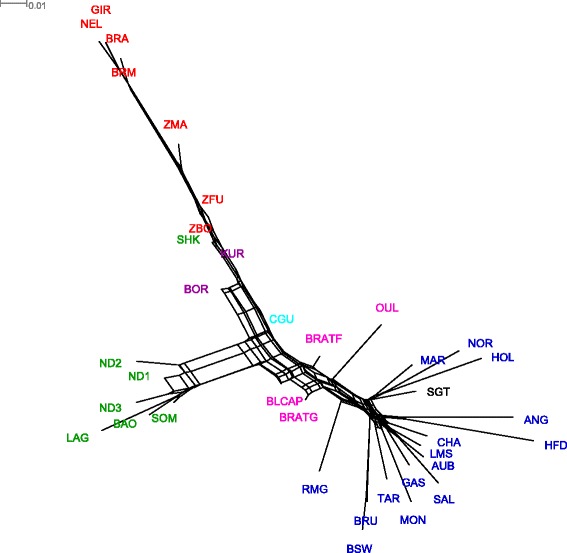
Neighbor net graph, constructed based on Nei’s genetic distance, relating the 37 populations of the study. Reticulations on the graph indicate past hybridization events between populations

To provide additional insight into the origin and evolutionary history of Tunisian populations, we constructed a NeighborNet graph based on Nei genetic distance (Fig. 4). Breeds belonging to one of the three main types (AFT, EUT and ZEB) were grouped together. In agreement with Fst and PCA results, Tunisian populations branched between AFT and EUT breeds with BLCAP closer than BRATG to AFT populations. Additionally, Tunisian populations showed short branch lengths indicating a low degree of divergence. Sets of parallel edges (reticulations) on the NeighborNet graph observed between BRATG and BLCAP indicate past hybridization events between the two populations.

**Fig 5:**
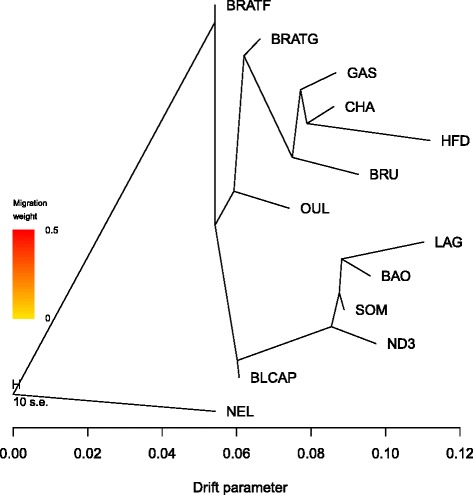
Maximum likelihood tree inferred from 13 cattle populations when no migration edges were fit. The scale bar shows ten times the average standard error of the estimated entries in the sample covariance matrix

**Fig 6:**
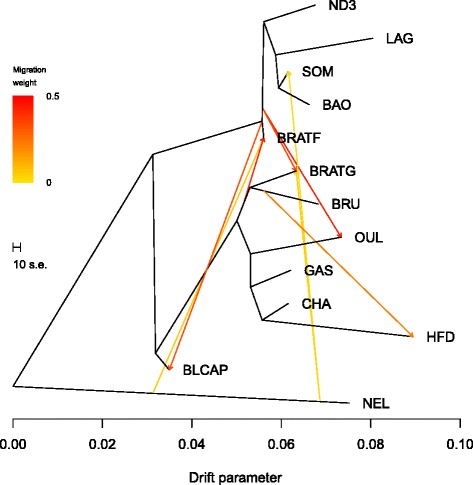
Maximum likelihood tree inferred from 13 cattle populations when eight migration events (modeled as arrows) were allowed. Migration arrows are colored according to their weight. The scale bar shows ten times the average standard error of the estimated entries in the sample covariance matrix

Finally, we used the Treemix software to model both population splits and gene flow between a subset of 13 populations. We first constructed a phylogenetic tree where no migration events were allowed (Fig. 5, residuals presented in Additional file 8). Among Tunisian populations, BRATG was the closest to European breeds while BLCAP was the closest to African populations. We then sequentially added up to eight migration events to the tree (Fig. 6, residuals presented in Additional file 9). To further check the topology of the phylogenetic tree and the consistency of migration edges, we performed two independent runs of Treemix with eight migrations (Additional file 10). We obtained roughly the same results in the three Treemix runs.

All the three phylogenetic trees showed BRATG sister to Brown Swiss (BRU) and identified high levels of introgression from the common ancestor of these two populations into BRATF (~45 %). Additionally, Treemix runs showed an important level of African cattle introgression into BRATG and OUL populations (~35 % and 44 %, respectively) and evidence of gene flow between BRATF and BLCAP (~34 %). Finally, low levels of indicine introgression were also identified into BRATF and BRATG (~9 % and 7 %, respectively). These results were in agreement with unsupervised hierarchical clustering results.

Significantly negative f3 statistics (see methods) showed evidence for AFT and EUT admixture in the three Tunisian populations with the strongest evidence for admixture found for BRATF (Additional file 11, Table S4 in Additional file 3). However, there was a clear difference in the origins of the ancestral European population between BLCAP and BRATG. In this regard, European ancestry of BLCAP derived mainly from North Europe (Three out of the five most significant tests) while that of BRATG derived mainly from Brown Swiss (Four out of the five most significant tests).

## Discussion

### Genetic diversity and relationship between Tunisian indigenous cattle

Our results show that gene diversity of the three Tunisian populations, measured by heterozygosity, was among the highest within the populations of the study and was similar to those observed in European breeds. This is more likely due to the combined effect of their European ancestry and their admixed origin. Indeed, all other hybrid populations with a European ancestry showed a similar range of genetic diversity.

We attributed low Hs values (relative to European breeds) within indicine breeds to ascertainment bias in the SNP discovery of the BovineSNP50 BeadChip. Indeed, in contrast to our findings, other studies showed that more diversity is observed within indicine breeds than within taurine breeds. For instance, the Bovine HapMap Consortium reported that nucleotide diversity in Brahman cattle is more than twice that observed within Holstein and Angus breeds [7, 8]. By analyzing sequence diversity assayed for 17 genes, Murray et al. [9] showed that nucleotide diversity is higher in indicine breeds than in European and African breeds, with the latter showing the lowest diversity for most of the sequenced genes [9]. The observed ascertainment bias led us to hypothesize that the genetic diversity of Tunisian populations is underestimated with the BovineSNP50 BeadChip as polymorphic sites of African origin present in the genome of Tunisian cattle were not included on the chip. The higher dispersion of Tunisian populations around their center of gravity in PCA and DAPC figures (Fig. 2, Additional files 4 and 6) and the neighbor-joining tree obtained from the genetic distance between each pair of individuals (Fig. 1), indicate higher genetic diversity in Tunisian populations than in European ones. This reflects the low levels of genetic drift (due to the absence of strong artificial selection) in Tunisian populations.

Low pairwise Fst values between Tunisian populations, shared ancestry detected between them in unsupervised hierarchical clustering when setting k to 37, and reticulations observed between Tunisian populations on the phylogenetic tree (Fig. 4), all suggest high levels of gene flow between the three populations. Moreover, pairwise Fst values, genetic distances between individuals, and Treemix phylogenetics all show that BLCAP and BRATF are genetically closer to each other than BRATG. This is probably due to the fact that the BRATG population is primarily isolated in the mountainous regions of northwest Tunisia, while the BLCAP and BRATF populations are located in closer geographic proximity in more accessible areas, thus leading to an easier mixing between these two populations.

### Tunisian local cattle breeds with two main ancestries

In this study, we presented consistent evidence of an admixed origin of Tunisian indigenous cattle with both African and European ancestries. Our analyses showed that these two ancestries are present in varying proportions between the three Tunisian populations. The difference is more pronounced between BLCAP and BRATG. At k = 3, the BLACP population tended to have a balanced proportion of African and European ancestries while BRATG had a more pronounced European influence. This hypothesis was corroborated by the results of pairwise population Fst, PCA, DAPC, and the phylogenetic analysis.

Increasing the number of inferred genetic clusters to five showed that European ancestry in Tunisian breeds arises both from North and South Europe, with the proportion of the latter being double of the former. This trend is supported by pairwise Fst results (lower differentiation between Tunisian populations/South European breeds than that between Tunisian populations/North European breeds) and by the proximity of Tunisian populations from South European rather than North European populations in the PCA and DAPC. Additionally, we noticed that the difference in the proportions of European ancestry between the three Tunisian populations arises from a South European origin. Previous genetic studies supported by archaeological evidence reported a difference between North and South European cattle populations, attributing it to movement of cattle occurring along two distinct routes from the cattle domestication center in the Middle East. The first migration route, called the Danubian route, was followed by Neolithic farmers as they moved along the Balkans into the plains of central and Northern Europe. The second, called the Mediterranean route, arose as farming spread from the Balkans and Southern Italy to Corsica, Southern France and Spain by 7700 years BP [10]. Based on these findings, we hypothesize that North European ancestry in Tunisian populations is old and could likely be traced back to the first domestication center in the Middle East, unlike South European ancestry whose influence on Tunisian populations is more recent.

From f3 statistics and the results of unsupervised hierarchical clustering (k = 7), we detected a substantial Brown Swiss influence, both on BRATF and BRATG and to a much lesser extent on BLCAP (only one significant f3 test indicated admixture between BLCAP and Brown Swiss). However, when Brune de l’Atlas populations were analyzed independently, we observed that only 12 % of the animals had more than 30 % of their ancestry originating from Brown Swiss, while more than 77 % of BRATF animals had less than 10 % of Brown Swiss ancestry (this level of admixture was similar to that observed within BLCAP individuals) and ~75 % of BRATG animals had less than 18 % of Brown Swiss ancestry. These findings led us to conclude that introgression from Brown Swiss occurred several hundreds of generations ago and left few footprints in the Brune de l’Atlas genome.

The pronounced signals of Brown Swiss identified in a few Brune de l’Atlas individuals most likely have a much more recent origin (i.e., a few generations ago). Indeed, due to their morphological similarity to Brown Swiss cattle, Brune de l’Atlas animals were commonly crossed with the Brown Swiss breed [11].

Likewise, from the low levels of genetic differentiation observed between Tunisian populations and the CGU breed, it has been posited that Tunisian populations are closely related to local breeds from the Iberian Peninsula. Indeed, CGU cattle were introduced in the Caribbean islands by Spanish and Portuguese conquerors after the second trip of Columbus in 1493 [12–14]. Many studies highlighted the North African taurine influence on Iberian cattle, which took place during two distinct migration events: the first during the Bronze Age [15, 16], and the second during the Moorish occupation from the 8th to the 13th century [17].

Additionally, our results suggest that African Taurine influence on Tunisian local cattle has a Longhorn origin and that the small amount of indicine ancestry found in the three populations mainly has an African origin. Archaeological studies reported that Longhorn Taurines were introduced from domestication centers (western Asia) into Africa through Egypt and the Horn of Africa in about 5000 BC [18], while zebu was introduced later into Africa from Arabia and Asia, through two migration waves: the first in around 1500 BC, where zebu cattle were crossed with Longhorn cattle in Ethiopia and Somalia, and the second during and after the Islamic conquests in around 670 BC [19]. Linking these reports to our results, we argue that indicine ancestry in Tunisian local cattle was inherited via African Taurine ancestors from the eastern and North-Eastern sides of the continent (i.e., present-day Ethiopia, Sudan, and Egypt) that spread west along with Arabic influences. Our hypothesis is corroborated by a recent study finding an increase in the percent of indicine ancestry when moving from west to east and from south to central Africa [20]. Additionally, we observed that, among the three Tunisian populations, BRATF showed the smallest degree of divergence. This was highlighted by shorter branch length in the phylogentic tree (both with Treemix and Splitstree), by lower pairwise Fst values with all breeds (compared to BLCAP and BRATG) and by greater evidence for admixture (strong negative Z-score when computing f3 statistics).

## Conclusions

To our knowledge, our study is the first of its kind aiming to assess the genetic structure of Tunisian indigenous populations and to establish their genetic origin using medium density SNP chips and comparisons with worldwide cattle. In the present study, we show that Tunisian local cattle populations have high genetic diversity, reflecting the absence of strong artificial selection. We also show the existence of a recent introgression of Brown Swiss in some Brune de l’Atlas individuals. Given that our sample collection took place at locations that are quite remote with respect to artificial insemination circuits, we can conclude that the presence of purely local individuals has become rare and thus highlighting the need to implement a national conservation strategy. Finally, as our study provided a comprehensive picture of the genetic structure and origins of Tunisian local cattle populations, it can also be used to investigate genetic variants underlying adaptation traits in these populations.

## Methods

### Animal ethics

All animal experimentation complied with the Tunisian Veterinary Authorities’ rules. No ethics approval was required by a specific committee, since the selected animals were not animals bred for experimental reasons.

### Selection of animals, blood sampling and genotyping

Blood samples belonging to 50 Tunisian animals from the three populations were collected from different geographical regions between the North and the center of the country. The BLCAP population is mainly found in the northeast of the country. Thus, most animals were selected from different geographical locations within this region. Similarly, Brune de l’Atlas (BRAT) individuals were sampled from both the northwestern and central parts of Tunisia (regions where most animals are located). Since most of the local animals were massively crossed with imported breeds, every attempt was made to sample purebred individuals. This was done by sampling the animals from areas that are isolated from the artificial insemination circuit. Many of these regions are located in mountainous areas.

Sampling was carried out by a team from the Livestock and Pasture Office of Tunisia (OEP). Identification of purebred animals was based on specific morphological criteria. Blood samples were collected in EDTA Vacutainer tubes. DNA was extracted using The Wizard® Genomic DNA Purification Kit (Promega) and by phenol-chloroform using a standard protocol [21]. DNA quantity and quality were evaluated on Nanodrop and agarose gel. DNA samples were then genotyped on the BovineSNP50 BeadChip Ver. 2 (Illumina, San Diego, CA, USA) using standard procedures (http://www.illumina.com) resulting in 52,886 autosomal genotyped SNPs.

We also included genotyping data from individuals belonging to 34 other cattle breeds (bovine Hapmap dataset) representative of European taurines (EUT, 15 breeds), African taurines (AFT, eight breeds), indicine (ZEB, seven breeds), and four crossbreed population (AFTXZEB, AFTXEUT, EUTXZEB and EUTXAFTXZEB). Genotyping data for 42,194 SNPs were available on these breeds, from three previous studies [22–24]. The number of animals per breed ranged from 14 to 30 (Additional file 12).

### SNP quality control and marker selection

We used PLINK ver.1.07 [25] (http://pngu.mgh.harvard.edu/~purcell/plink/) for genotyping data quality control. Samples genotyped for less than 90 % of markers were excluded from the analysis. SNPs genotyped for less than 90 % of the animals and those with MAF less than 0.01 were also discarded.

Using these criteria, we excluded one individual from the BRATG population due to a low genotyping rate (<90 %). Similarly, 1115 SNPs were excluded because of low genotyping rates, and 5949 SNPs were also excluded as they were monomorphic (MAF <0.01). An exact test for Hardy-Weinberg Equilibrium (HWE) was then carried out within each population separately on the remaining SNPs using PLINK, which led to 45,691 SNPs retained for further analysis.

Genotyping data from Tunisian samples were then merged with the bovine Hapmap dataset. The whole dataset consisted of 878 individuals from 37 populations genotyped for 38,597 SNPs spread over all autosomal chromosomes. Average marker density was one SNP every 65.6 kb (Table S1 in Additional file 3).

### Analysis of population structure

We used various methods to provide a fine-scale assessment of the genetic structure of Tunisian populations. First we computed the genetic distance for each pair of individuals using the ape R package [26]. The distance between two individuals was defined as the number of loci for which they differ. A neighbor-joining tree was then computed based on the resulting distance matrix using the phyclust R package [27]. Then a PCA was performed with all the available 38,597 SNPs using the adegenet R package [28]. In addition, we used DAPC implemented in the adegenet R package [29] based on all the available SNP information to provide further details about the genetic structure of Tunisian populations. DAPC is a non model-based method using PCA as a prior step. It provides a description of genetic clusters using a few discriminant functions. This method identifies an optimal number of genetic clusters that best describe the data by running a k-means clustering algorithm and comparing the different clustering solutions using the Bayesian Information Criterion (BIC).

In our DAPC analysis, we first determined the optimal number of retained principal components using the function optim.a.score implemented in adegenet. We retained 60 principal components that cumulatively explained 40 % of the total variance of the data. Finally, we performed a first run of DAPC with an a priori assignment of individuals to their population of origin. This analysis, which is referred to as the predefined DAPC group, provided membership probabilities of each individual for the populations of the study. This information was used to check if Tunisian animals were correctly assigned to their predefined populations. Then a second DAPC analysis was run using k-means and identified the optimal number of genetic clusters. Individuals were then assigned to these clusters. This analysis is referred to as a posteriori DAPC.

Unsupervised hierarchical clustering was carried out using the Admixture 1.23 software [6]. Distruct software [30] was then used to graphically display ancestry within each individual.

### Genetic diversity and population differentiation analysis

We used expected heterozygosity (Hs) and inbreeding within subpopulations measured by Fis to assess the genetic diversity of each population.

In order to check for an effect of ascertainment bias (due to SNP discovery) on genetic diversity parameters, we estimated (Hs) using either all data (38,597 SNPs) or only SNPs with MAF above 0.01 in African and indicine populations. Discarding SNPs whose MAF was below 0.01 is supposed to limit the effect of ascertainment bias.

Genetic differentiation between populations was estimated using Genepop 4.0 software [31] using all available SNPs. The R-package StAMMP [32] was used to estimate a 95 % confidence interval (CI) of pairwise Fst based on 5000 bootstraps. The R-package hierfstat [33] was used to construct a 95 % CI of inbreeding coefficient, measured by Fis, for all populations using a subset of 5000 randomly chosen SNPs and 100000 bootstraps.

### Phylogenetic analysis

Phylogenetic relationships between populations were investigated using Nei genetic distance. NeighborNet graphs were constructed from the estimated genetic distances using the Splitstree software [34].

Patterns of splits and mixtures of a subset of 13 populations were carried out using Treemix [35] and by setting Nelore (NEL) as a rooting outgroup. First, we built a maximum likelihood tree of the populations with no migration events allowed. Then, we constructed a phylogenetic network for all the selected populations, thereby increasing migration events (modeled as edges) sequentially up to eight migrations. To further evaluate the consistency of migration edges, we ran TreeMix two separate times with eight migration events. The residuals from the fit of the model to the data were visualized using the R script implemented in Treemix. Finally, in order to provide further support for a past admixture between populations, we ran the THREEPOP program implemented in Treemix. This program calculated f3 statistics for all possible triplets from the selected populations. If a population A is a mixture of two other populations B and C, the Z-score computed for each tested triplet would have a significant negative value.

### Availability of supporting data

The genotyping data and the phylogenetic trees supporting the results of this article are available through the Data Dryad digital repository. doi:10.5061/dryad.sj548.

## Additional files

Additional file 1:
**Genetic diversity parameters for the 37 cattle populations.**
Additional file 2:
**Minor Allele Frequencies (MAF) Distribution of 38,597 SNPs in African (AFT), European (EUT) and indicine (ZEB) populations.** The figure shows higher proportions of SNPs with rare variants (MAF <0.1) within African and Indicine populations than European breeds. Additional file 3:
**This additional file is an excel file containing 4 sheets each corresponding to the following supplementary tables: Table S1: Description of SNP distribution per chromosome.** Table S2: Fis 95 % confidence interval (CI) and Fst values for each pair of populations estimated using the R software. Fis 95 % CI (cells highlighted in green) were estimated with the R package hierfstat from 5000 randomly chosen SNPs and 100,000 bootstraps. Fst between pairs of populations were estimated with the R package StAMPP. 95 % CI intervals are written between brackets and were obtained using 38,597 SNPs and 5000 bootstraps. Table S3: Fis (cells on the diagonal highlighted in green) and Fst values for each pair of populations estimated using Genepop software. Table S4: Fifty most significant f3 statistics for the subset of 13 populations used in Treemix analysis. Additional file 4:
**Predefined group DAPC of allele frequencies obtained from 38,597 SNPs genotyped in 878 cattle individuals from 37 populations.** To avoid the risk of overfitting the discriminant functions, 60 principal components that cumulatively explained 40 % of the total variance of the data were retained. The optimal number of principal components was determined using the function optim.a.score implemented in adegenet. Additional file 5:
**Membership probability of the 49 Tunisian individuals.** These probabilities were derived from the predefined groups from DAPC. Each bar indicates the probability of assignment of an individual to a population. Only membership probabilities above 0.95 were reported. Additional file 6:
**A posteriori DAPC results. In this analysis, the optimal number of genetic clusters was determined by the adegenet package.**
Additional file 7:
**Differentiation, measured by Fst, of Tunisian populations versus the other populations of the study.**
Additional file 8:
**Plot of residuals from the maximum likelihood tree obtained when no migration edges were fit (Fig.** 5 **).** Colors are described in the palette on the right. Additional file 9:
**Plot of residual from the maximum likelihood tree obtained when 8 migration edges were fit (Fig.** 6 **).** Colors are described in the palette on the right. Additional file 10:
**Two replicates of the phylogenetic network with eight migration edges.** The aim is to evaluate consistency of migration edges fit in Fig. 6. Migration arrows are colored according to their weight. The scale bar shows 10 times the average standard error of the estimated entries in the sample covariance matrix. Additional file 11:
**Five most significant f3 statistics for BLCAP, BRATG and BRATF admixture.**
Additional file 12:
**Sample description: the number of individuals, the type (EUT = European taurines, AFT = African Taurines, ZEB = Zebu) and the data origin of each population.**

## Abbreviations

BAO: Baoule
LAG: Lagune
ND1: N’Dama
ND2: N’Dama
ND3: N’Dama
SHK: Sheko
SOM: Somba
BOR: Borgou
KUR: Kuri
ANG: Angus
AUB: Aubrac
BRU: French Brown Swiss
BSW: Brown Swiss from USA
CHA: Charolais
GAS: Gascon
HFD: Hereford
HOL: Holstein (FR)
LMS: Limousin
MAR: Maraichine (Parthenaise)
MON: Montbeliarde
NOR: Normande
RMG: Romagnola
SAL: Salers
TAR: Tarine
BLCAP: Blonde du Cap Bon
BRATF: Brune de l’Atlas Fauve
BRATG: Brune de l’Atlas Grise
OUL: Oulmès Zaer
CGU: Creole from Guadeloupe
SGT: Santa Gertrudis
BRA: Brahman from Martinique
BRM: Brahman from USA and Australia
GIR: Gir
NEL: Nelore
ZBO: Zebu Bororo
ZFU: Zebu Fulani
ZMA: Zebu from Madagascar.
SNP: Single Nucleotide Polymorphism
EUT: European Taurines
AFT: African Taurines
ZEB: Zebu
MAF: Minor Allele Frequency
HWE: Hardy-Weinberg Equilibrium
PCA: Principal Components Analysis
DAPC: Discriminant Analysis of Principal Components
BIC: Bayesian Information Criterion
PC: Principal Component
