# Supplementary figures and images for: Genome-wide insights into population structure and genetic history of tunisian local cattle using the illumina bovinesnp50 beadchip
Source: BMC Genomics. 2015 Sep 4;16(1):677. doi: 10.1186/s12864-015-1638-6 (PMC4560074; doi:10.1186/s12864-015-1638-6)

MAF distribution in AFT, EUT and ZEB populations (38597 SNPs)

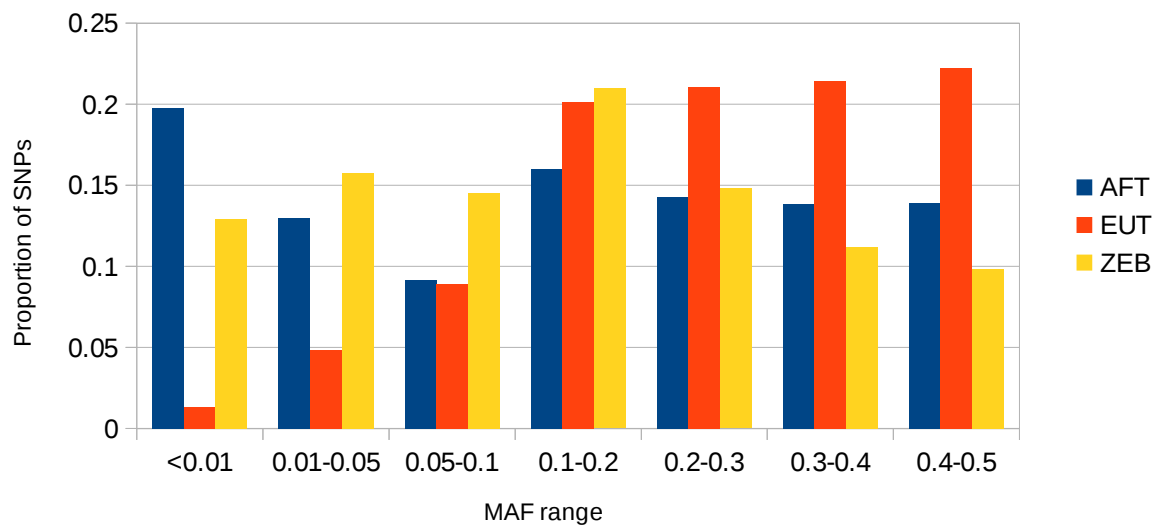

Supplement: Additional file 2: — Minor Allele Frequencies (MAF) Distribution of 38,597 SNPs in African (AFT), European (EUT) and indicine (ZEB) populations. The figure shows higher proportions of SNPs with rare variants (MAF <0.1) within African and Indicine populations than European breeds. [file 12864_2015_1638_MOESM2_ESM.pdf]

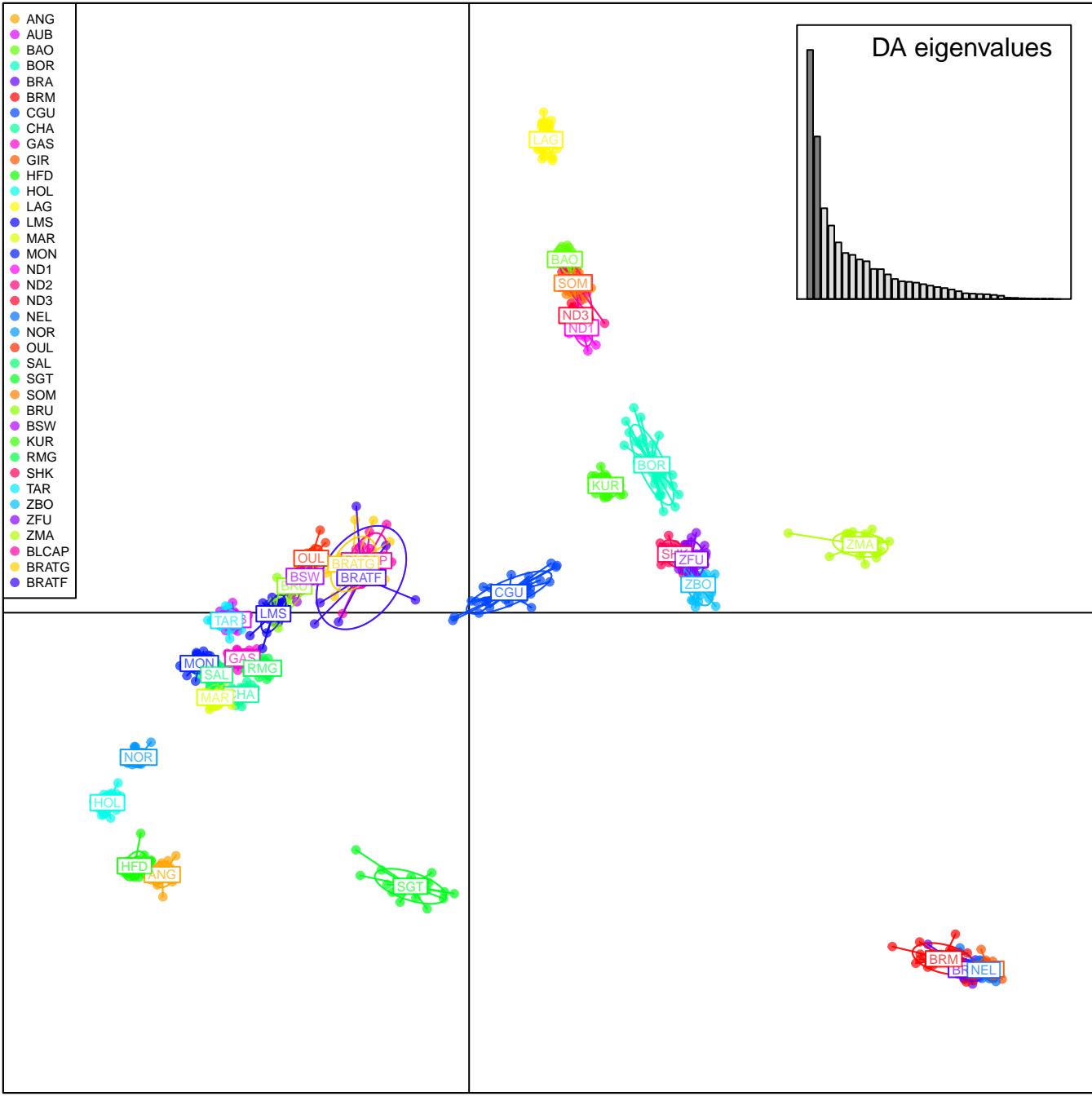

Supplement: Additional file 4: — Predefined group DAPC of allele frequencies obtained from 38,597 SNPs genotyped in 878 cattle individuals from 37 populations. To avoid the risk of overfitting the discriminant functions, 60 principal components that cumulatively explained 40 % of the total variance of the data were retained. The optimal number of principal components was determined using the function optim.a.score implemented in adegenet. [file 12864_2015_1638_MOESM4_ESM.pdf]

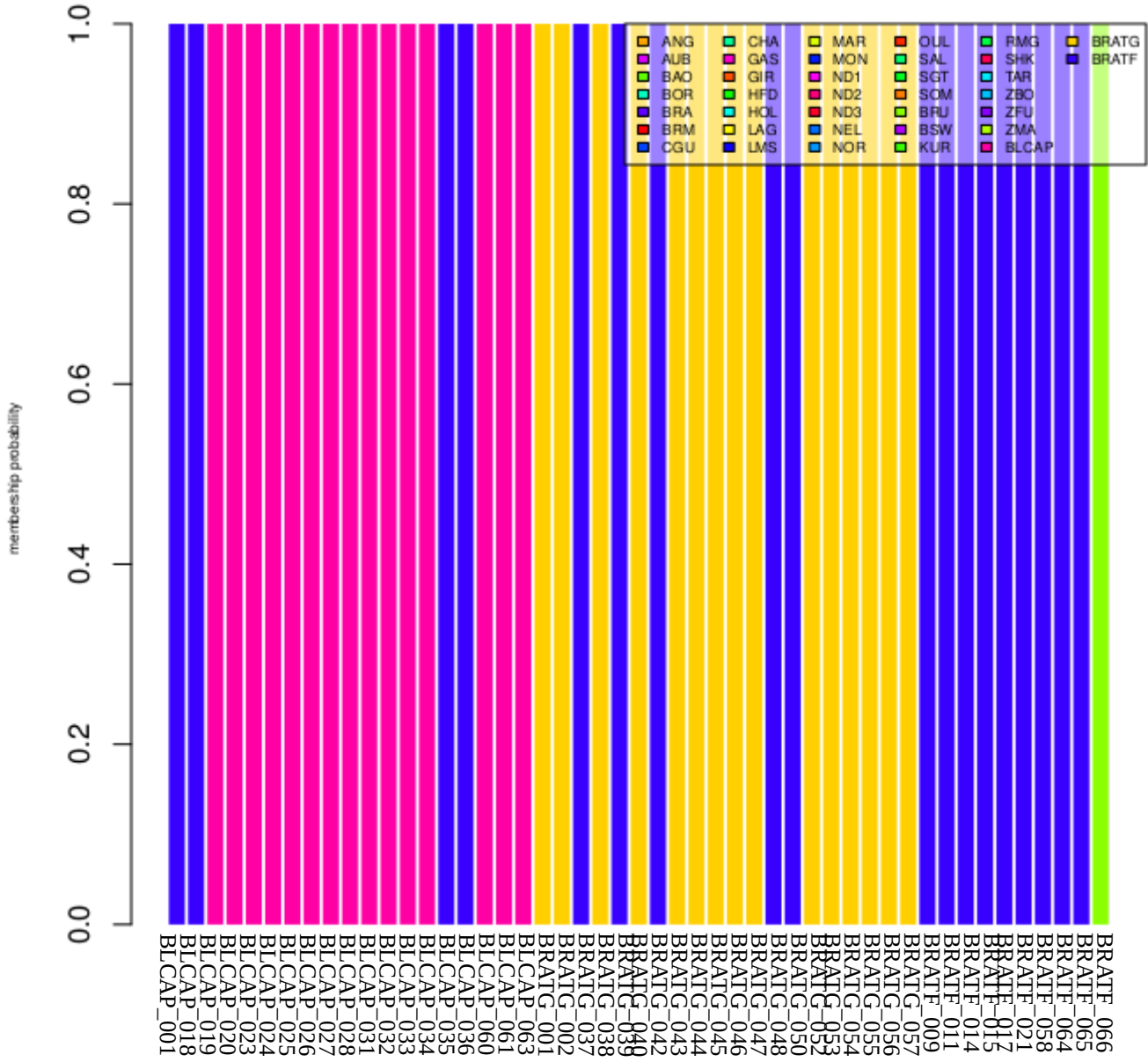

Supplement: Additional file 5: — Membership probability of the 49 Tunisian individuals. These probabilities were derived from the predefined groups from DAPC. Each bar indicates the probability of assignment of an individual to a population. Only membership probabilities above 0.95 were reported. [file 12864_2015_1638_MOESM5_ESM.pdf]

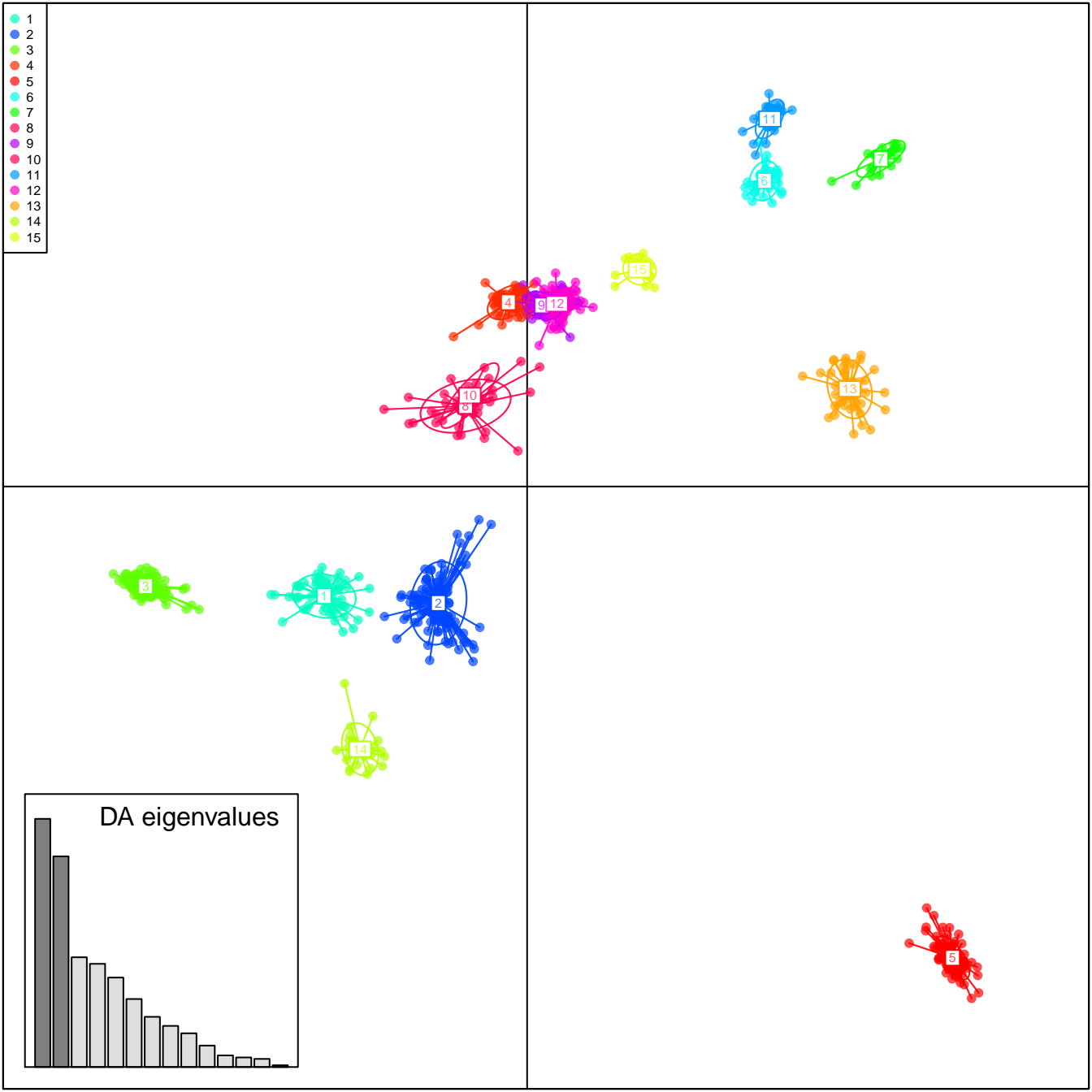

Supplement: Additional file 6: — A posteriori DAPC results. In this analysis, the optimal number of genetic clusters was determined by the adegenet package. [file 12864_2015_1638_MOESM6_ESM.pdf]

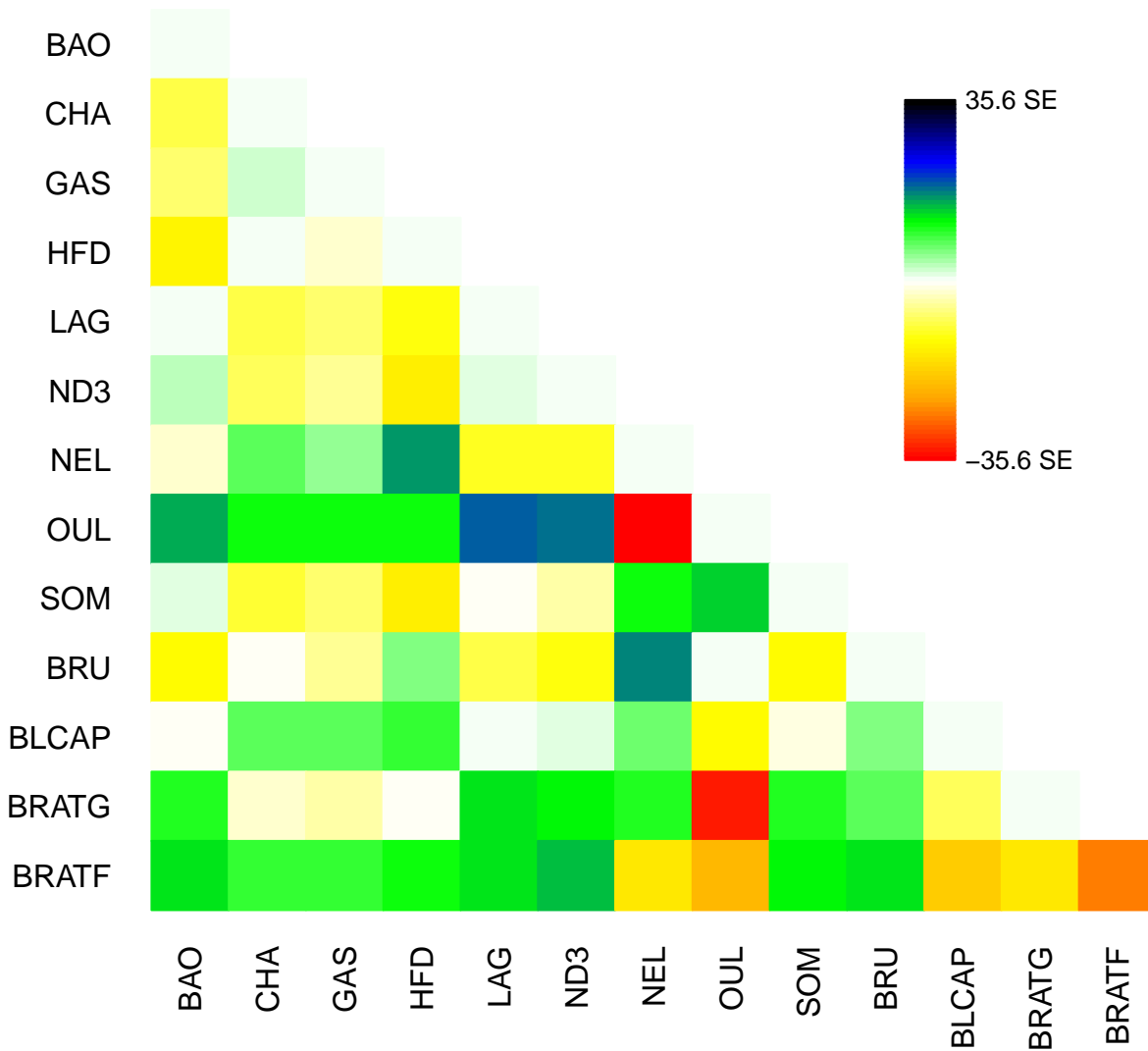

Supplement: Additional file 8: — Plot of residuals from the maximum likelihood tree obtained when no migration edges were fit (Fig. 5 ). Colors are described in the palette on the right. [file 12864_2015_1638_MOESM8_ESM.pdf]

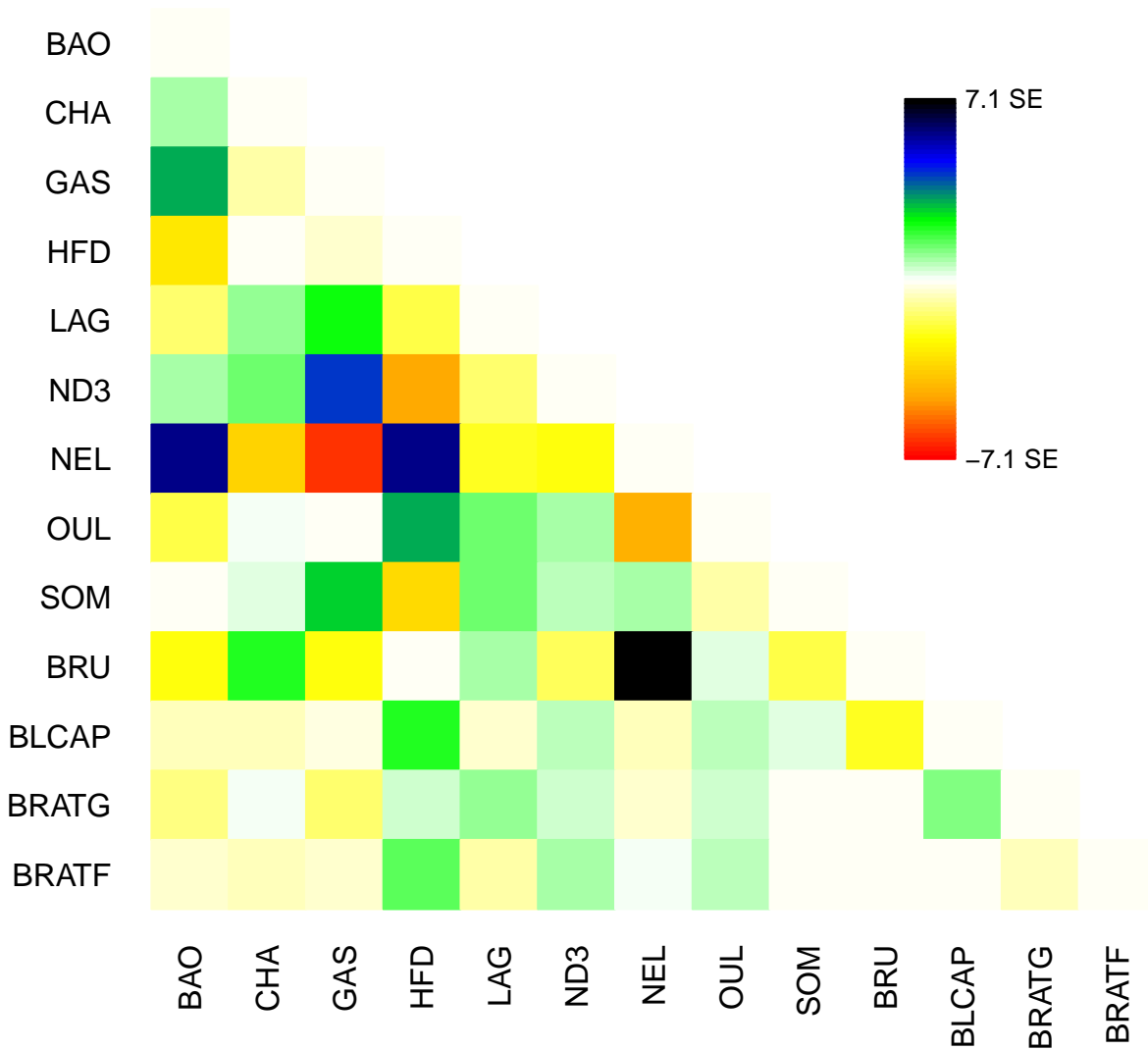

Supplement: Additional file 9: — Plot of residual from the maximum likelihood tree obtained when 8 migration edges were fit (Fig. 6 ). Colors are described in the palette on the right. [file 12864_2015_1638_MOESM9_ESM.pdf]

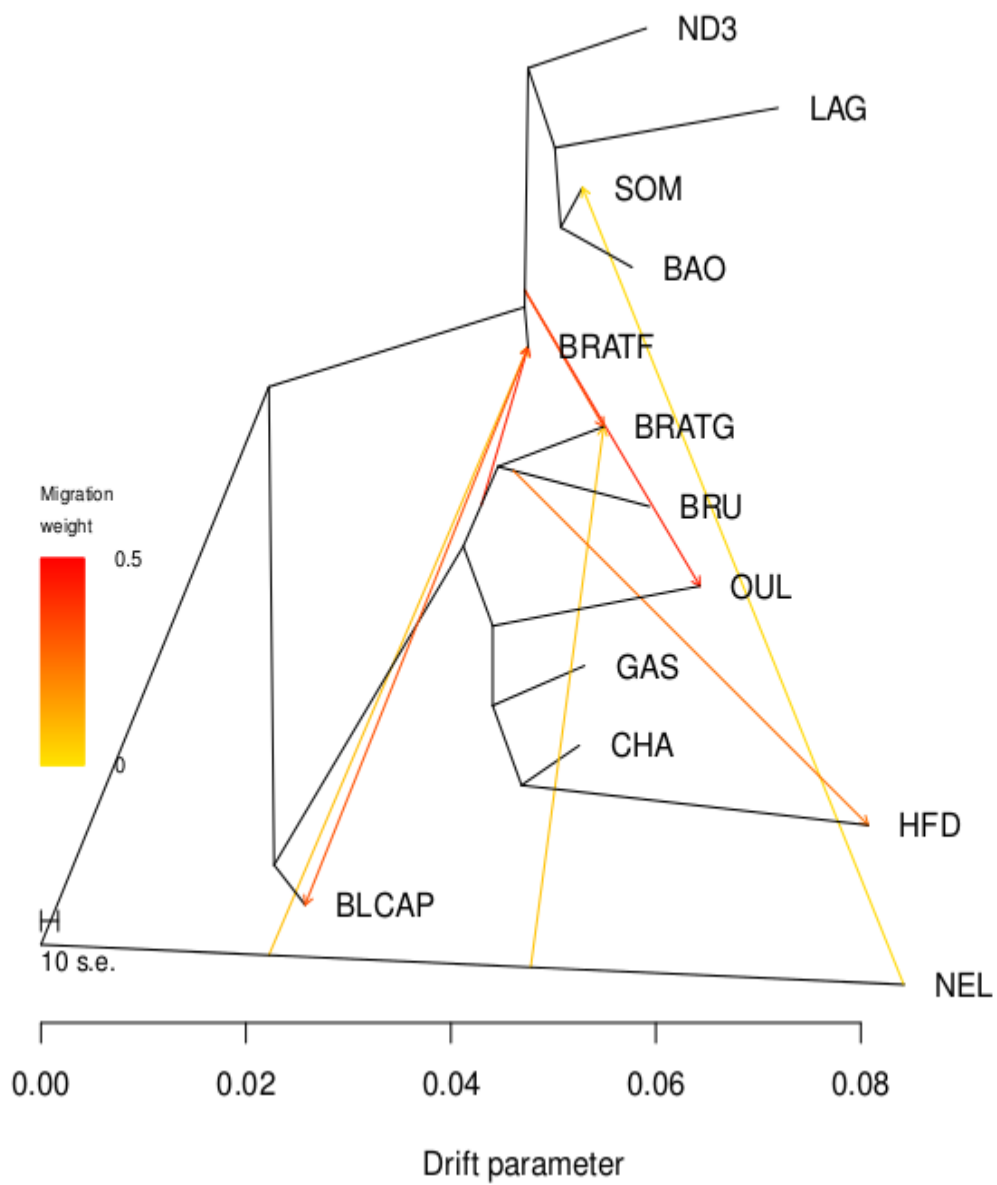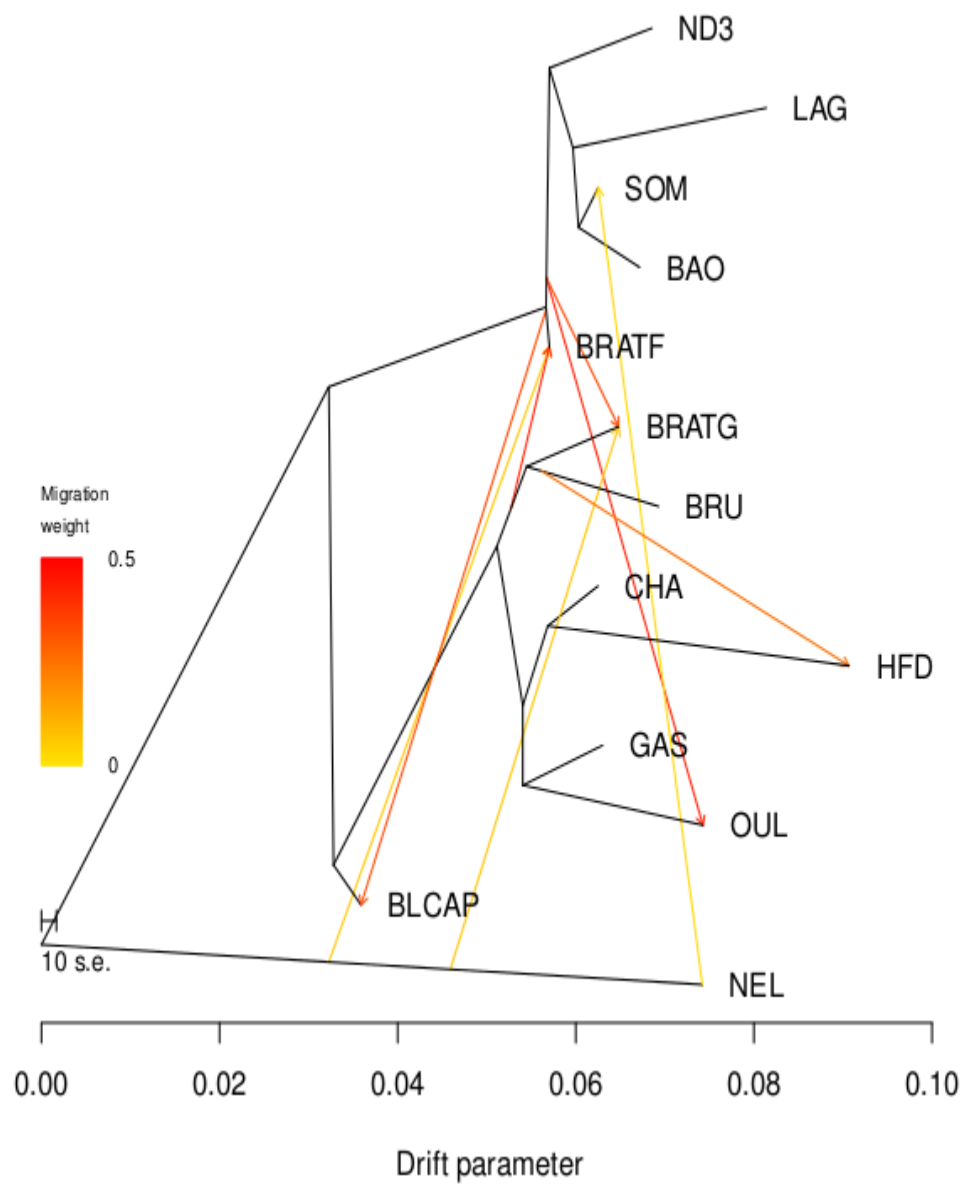

Supplement: Additional file 10: — Two replicates of the phylogenetic network with eight migration edges. The aim is to evaluate consistency of migration edges fit in Fig. 6. Migration arrows are colored according to their weight. The scale bar shows 10 times the average standard error of the estimated entries in the sample covariance matrix. [file 12864_2015_1638_MOESM10_ESM.pdf]
